# An Association between Initiation of Selective Serotonin Reuptake Inhibitors and Suicide - A Nationwide Register-Based Case-Crossover Study

**DOI:** 10.1371/journal.pone.0073973

**Published:** 2013-09-09

**Authors:** Charlotte Björkenstam, Jette Möller, Gunilla Ringbäck, Peter Salmi, Johan Hallqvist, Rickard Ljung

**Affiliations:** 1 Department of Public Health Sciences, Karolinska Institutet, Stockholm, Sweden; 2 Department of Statistics, Monitoring, and Evaluation, National Board of Health and Welfare, Stockholm, Sweden; 3 Department of Public Health and Caring Sciences, Uppsala University, Uppsala, Sweden; 4 Department of Molecular Medicine and Surgery, Stockholm, Sweden; University of British Columbia, Canada

## Abstract

**Background:**

Treatment with selective serotonin reuptake inhibitors (SSRI) is one of the most common treatments for depression. It is however not clear whether or not there is an increased short-term suicide risk during initiation with SSRI.

**Methods:**

A register-based nationwide case-crossover study including 5,866 suicides, 1,698 women and 4,168 men, from the Death Register 2007-2010 in Sweden. SSRI initiation was defined as a dispensed prescription of SSRI within 28 days prior to the date of suicide with no previous dispensed prescription of SSRI within 4 months prior that prescription. The control period took place one year earlier. Odds ratio (OR) was estimated using conditional logistic regression.

**Result:**

During the 28 day period prior to suicide 48 women and 138 men were exposed to SSRI initiation (while not being exposed in the control period) and 22 women and 43 men were exposed in the control period (while not being exposed in the case period). The OR for suicide after initiation with SSRI was 2.7 (95% CI: 1.6-44) for women, and 4.3 (95% CI: 3.0-6.1) for men. The highest OR was found 8-11 days after initiation with SSRI 9.7 (95% CI: 3.0-31.7) for women and men combined.

**Conclusion:**

The main limitation in this study is confounding by indication, but the descriptive question is however not confounded by indication. Together with plausible biological mechanisms and previous clinical and epidemiological observations our findings, linking initiation of SSRI to increased short-term suicide risk, deserve further attention specifically in the clinical setting.

## Introduction

It is not clear whether or not there is an increased short-term suicide risk during initiation of therapy with SSRI [1–11].

In a case-control study of the UK General Practice Research Database the relative risk for suicide was 38 times higher 1–9 days after prescription of an antidepressant than during an unexposed time period [7]. In a meta-analysis of 372 double blind randomized placebo controlled trials with 99 231 adults assigned to SSRI or placebo showed an initial increased suicide risk immediately after initiation of SSRI among patients under age 25 but no increased risk among the oldest, aged 65 and above [9]. Other studies have also shown an increased risk of suicide after initiation of SSRI in adolescents and young adults [5,12]. At initiation with SSRI therapy, depressed patients might experience a so called activation syndrome or behavioral disinhibition, characterized by irritability, panic attacks, anxiety, agitation, insomnia, and hostility, where suicidal thoughts may follow or increase in the early stages of treatment before the mood improvement provided by the treatment has an effect [6,10]. It is estimated that around 4% of patients initiated on SSRI therapy develop activation syndrome [10]. The mechanism between SSRI therapy and activation syndrome has not been established yet but it has been hypothesized that anti-depressants improve patient energy levels before they improve mood, which may contribute to the increase in risk of suicide during the early stages of treatment [8]. However, the specific role of serotonin in functions such as impulsivity and aggression may provide a possible biological mechanism where SSRI therapy more specifically might trigger suicide in some individuals [13]. The plausible biological mechanisms together with clinical and epidemiological observations linking initiation of SSRI to increased short-term suicide risk deserve further attention.

Therefore we conducted a large nationwide register-based case-crossover study to explore the short-term risk of suicide after SSRI initiation, whether the risk of violent suicide is more increased than the risk of non-violent suicide, and finally, whether the risk differs by age and sex.

## Method

### Ethics statement

The study population was based on linkage of several public national registers. Ethical vetting is always required when using register data in Sweden. The ethical vetting is performed by regional ethical review boards and the risk appraisal associated with the Law on Public Disclosure and Secrecy is done by data owners. The ethical review boards can however waive the requirement to consult the data subjects (or in case of minors/children the next of kin, careers or guardians) directly to obtain their informed consent, and will often do so if the research is supported by the ethical review board and the data has already been collected in some other context. According to these standards in Sweden this project has been evaluated and approved by the Regional Ethical Review Board of Karolinska Institutet, Stockholm, Sweden.

#### Case-crossover design

We performed a register-based nationwide case-crossover study. The case-crossover design was introduced in 1991 by Maclure [14]. The method is partly similar to a matched case-control study. Though, instead of comparing with other individuals the case acts as its’ own control. The method is preferably used when exposure causes a transient change in risk of a disease with acute onset [14]. The induction time, that is, the time between exposure and the outcome, is assumed to be short, hours or days rather than years. If an exposure has a triggering effect, it should be more frequent in the period prior outcome, than in a period more distant and without outcome. The exposure frequency during the á priori time period before the event i.e. the *case period* is compared to the exposure frequency during one or more *control periods* for the same individual.

#### Study population

We identified 5 913 individuals aged 13 years or older who had committed suicide between 2007 and 2010 as recorded in the Swedish Causes of Death Register. We chose to include young individuals i.e. from age13 since earlier studies have found increase in suicide risk among the very youngest [9,12]. We included deaths coded as suicide according to the *International Classification of Diseases* (ICD-10: X60-X84) and to avoid changes and trends in classification we also included deaths coded as undetermined intent (ICD-10: Y10-Y34) [15]. The Causes of Death Register contains information on all deceased Swedish residents since 1952 and has a very high coverage, though for 0.5% there is a lack of medical information regarding the cause of death [16].

We linked our cases with the Swedish Prescribed Drug Register to obtain all prescriptions of SSRI (according to the Anatomical Therapeutic Chemical (ATC) classification: N06AB) between July 2005 and the 31^st^ of December 2010.

The Swedish Prescribed drug register contains information on all prescribed drugs dispensed at a pharmacy in the entire population of Sweden. However, the register does not include data on drugs given during hospitalizations [17]. The register keeps information about the date of dispensing as well as dispensed drug formulation, strength and quantity.

Patients with recent psychiatric hospitalizations (ICD-10: F00-F99) i.e. hospitalizations within one month before the dispensed prescription during both the case and the control period were excluded since they might have initiated SSRI therapy at the hospital. These data were obtained from the National Patient Register, which contains data for all discharges from Swedish hospitals since 1987.

#### Exposure in the case period

**Figure 1:**
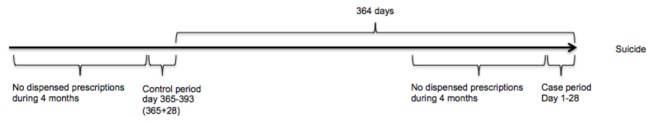
Graphical presentation of the definitions of case and control periods.

Date of suicide was considered as the index date. If a case had been hospitalized for self-destructive behaviour and was subsequently discharged as dead, we considered the date of hospitalization as the index date instead. This was done to capture the actual date of a severe suicide attempt leading to suicide, irrespective whether the patient died immediately or after some days. Exposure to SSRI initiation was defined as a dispensed prescription of SSRI within 28 days prior to the index dates with no previous dispensed prescription of SSRI within 4 months prior that prescription (Figure 1). According to the regulations for pharmaceutical benefits under the national health plan, a patient is not to receive more medication on any one occasion than they are expected to need for the next 90 days. Hence, we chose four months in an attempt to select only initiations. In a sensitive analysis we extended this time period to six months.

Individuals who filled prescription of SSRI within 28 days prior to index date and with at least one previous filled prescription within 4 months prior to that prescription were consequently *not* regarded as initiating SSRI therapy.

We regarded a hospitalization for any psychiatric condition as a possible indication of SSRI treatment. Hence, patients discharged within one month prior to the case period, not in connection with the index event, were not regarded as exposed to SSRI initiation. This solely because information on drugs given during in-patient care is not included in the Prescribed Drug Register and was therefore not available. One month was chosen since you can get SSRI at the hospital for the first couple of days and hence do not need a prescription.

#### Exposure in the control period

We defined the index date in the control period 364 days prior to the suicide in order to avoid possible seasonal differences and to ensure that the index date in the case period and in the control period occurred on the same weekday. Being exposed in the control period was defined in the same way as for the case period, i.e. as a dispensed prescription of SSRI within 28 days prior to the index date (364-392 days before suicide) with no previous dispensed prescription of SSRI within 4 months prior that prescription. Hospitalization for any psychiatric condition was also considered in the same way as for the case period.

#### Statistical analysis

We used a case-crossover design with the matched-pair interval approach (using control information based on the matched-pair control period) analyzed by conditional logistic regression. The odds ratio (OR) represents the odds of SSRI initiation during the period prior to suicide compared to the odds of SSRI initiation in the control period (one year earlier). The ORs are considered estimates of the incidence rate ratio comparing the risk of suicide in exposed time-periods to the suicide risk in unexposed time periods [18]. All analyses used were planned before any data collection, and no prior inspection of the relevant data from these registries was obtained prior to designing the study. In the analyzes we á priori categorized the case period into 7 different hazard periods; 0-3 days prior the suicide, 4-7 days, 8-11, 12-15, 16-19, 20-23, and 24-28.

In a sub analysis we stratified the suicides as certain suicides (X60-X84) and undetermined intent (Y10-Y34) respectively. We also dichotomized suicides as violent (X70-X84; Y20-Y34) and non-violent (X60-X69; Y10-Y19) ) and all other methods were regarded as violent (ICD-10: X70-X84; Y20-Y34). In sensitivity analyses we used a six months SSRI free window prior to initiation instead of four months. To validate our results we performed another analysis according to the same principles as our main analysis but with tricyclic antidepressants (TCA, according to the Anatomical Therapeutic Chemical classification: N06AA) instead of SSRI. This was done as a comparison in an attempt to value the potential effect of SSRI.

SAS PHREG procedure was used to calculate odds ratios and 95% confidence intervals (CI). SAS Enterprise Guide 4.2 (SAS Institute Inc, Cary, NC, USA) was used.

## Results

The analyses included 5 913 suicides, whereof 1 711 (29%) women and 4 202 (71%) men. Mean age at time of suicide among women was 51 (SD:18) and was 50 (SD:18) among men. During the 28 days prior to suicide 59 (3.4%) women and 169 (4.0%) men were exposed to initiation of SSRI therapy while not being exposed during the control period. During the corresponding time period one year earlier 22 women (1.3%) and 41 (1.0%) men were exposed to initiation of SSRI therapy (while not being exposed during the case period) (table 1).

**Table 1:** Number of exposed (Exp) and unexposed (Unexp) during the case period and the control period respectively (case period: control period) during different initiation days, by sex.

	**Women**							**Men**			
**Initiation days**	Exp:Exp*	Exp:Unexp	Unexp:Exp	Unexp:Unexp	OR 95% CI		Exp:Exp	Exp:Unexp	Unexp:Exp	Unexp:Unexp	OR 95% CI
0-28	0	56	23	1 619	2.43 (1.50-3.95)		0	163	43	3 962	3.93 (2.79-5.53)
0-2	0	8	3	1 687	2.66 (0.71-10.04)		0	14	4	4 150	3.50 (1.15-10.63)
3-4	0	4	3	1 691	1.33 (0.30-5.94)		0	16	6	4 146	2.66 (1.04-6.81)
5-7	0	9	2	1 687	4.50 (0.97-20,82)		0	28	5	4 135	5.60 (2.16-14.50)
8-11	0	7	1	1 690	6.99 (0.86-56.76)		0	21	1	4 146	20.95 (2.82-155.36)
12-14	0	4	2	1 692	2.00 (0.37-10.92)		0	14	3	4 151	4.67 (1.34-16.24)
15-20	0	16	2	1 680	8.00 (1.84-34,79)		0	22	7	4 139	3.14 (1.34-7.36)
21-28	0	8	10	1 680	0.80 (0.32-2.03)		0	48	17	4 103	1. %2 1.62-4.91)

* The case period is represented to the left of the colon and the control period on the right. The first column displays individuals exposed in both the case period and in the control period, Exp:E

**Figure 2:**
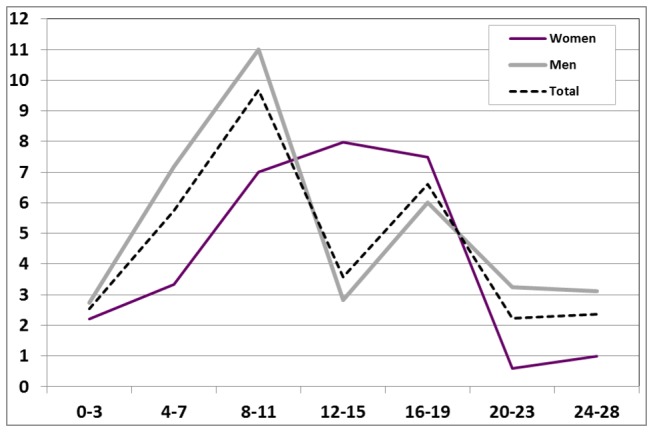
Odds ratios for suicide, number of days after initiation of SSRI.

We found an overall increased risk of suicide during the first 28 days of initiation of SSRI therapy with an OR of 3.7 [95% CI: 2.8-4.9] (table 2). Women displayed a slightly lower effect estimate than men with an OR of 2.7 [1.6-4.4] whereas the OR among men was 4.3 [3.0-6.1]. Induction time analyses showed the overall highest risk in the 8-11 day period after SSRI initiation with an overall OR of 9.7 [2.9-31.7] (Figure 2) and with sex specific OR for men of11.0 [2.6-46.8], and for women an OR of 7.0 [0.9-56.8]. Women displayed the highest OR during days 12-15 with OR 8.00 [1.0-63.7]. During the last week (day 24-28) the OR among women was reduced to 1.0.

**Table 2:** Odds ratios for suicide during 28 days of initiation with SSRI, stratified by age and sex.

	**Total**	**Women**	**Men**
**Age group**	**OR (95% CI)**	**OR (95% CI)**	**OR (95% CI)**
Total	2.92 (2.19-3.89)	2.43 (1,50-3,95)	3.93 (2.79-5.53)
13-25	2.29 (0.94-5.55)	1.25 (0.37-4.66)	3.67 (1.02-13.14)
26-44	2.56 (1.44-4.56)	2.60 (0.93-7.29)	2.54 (1.27-5.11)
45-64	3.44 (2.24-5.29)	2.25 (0.98-5.17)	3.95 (2.39-6.53)
65-74	4.13 (1.91-8.93)	10.0 (1.28-78.11)	3.29 (1.41-7.66)
75+	5.67 (2.38-13.49)	2.0 (0.68-5.85)	24.0 (3.25-177.40)

The odds ratio of suicide during the first four weeks of initiation of SSRI therapy seemed to slightly increase with age although not statistically significant (table 2). When we extended the dispensed prescription-free time window from 4 to 6 months, this generated similar results as the main analysis (data not shown).

When we restricted our analysis to cases classified as violent suicides, the overall OR for suicide during the first four weeks of initiation of SSRI therapy was 4.8 [3.2-7.2] for men and 3.2 [1.6-6.3] for women (table 3). For women and men combined the OR for violent suicide during the 8-11 day period after SSRI initiation was 19.0 [2.5-141.5 separately] (data not shown). In a sub analysis studying certain suicides and death of undetermined intent separately, the ORs were 4.1 [3.0-5.7] and 1.5 [0.8-7.7], respectively (table 3). The OR for certain suicides for the 8-11 day period was 21.9 [3.0-162.2] (data not shown).

**Table 3:** Odds ratios for violent, non violent suicides, certain suicides and deaths with undetermined intent during 28 days of initiation with SSRI.

	**Total**	**Women**	**Men**
**Type of death**	**OR (95% CI)**	**OR (95% CI)**	**OR (95% CI)**
Violent	4.32 (3.03-6.14)	3.18 (1.62-6.27)	4.78 (3.16-7.23)
Non violent	2.04 (1.28-3.26)	1.75 (0.86-3.56)	2.29 (1.22-4.28)
Certain suicides	4.13 (2.99-5.70)	3.92 (2.08-7.38)	4.21 (2.89-6.11)
Undetermined intent	1.50 (0.83-7.72)	0.82 (0.34-1.98)	2.57 (1.07-6.15)
Certain suicides			
violent	4.59 (3.17-6.65)	4.13 (1.91-8.93)	4.73 (3.10-7.22)
- non violent	2.83 (1.47-5.47)	3.50 (1.15-10.63)	2.50 (1.10-5.67)

## Discussion

In our study of 5 913 suicides, we found an increased suicide risk after initiation of SSRI therapy. The risk increase was highest in the beginning of the second week of treatment, day 8-11 for men and during days 12-15 among women. In contrast to previous studies we did not find the suicide risk increase to be higher among young adults.

The main strengths of the present study include the large sample size, the population-based and complete nationwide coverage of all suicides and of all filled prescriptions of SSRI. The nationwide register-based design reduces possible recall bias and selection bias. Moreover, the case-crossover design with the matched-pair interval approach where all cases act as their own controls eliminates confounding by variables not varying over short time periods e.g. age, gender, ethnicity, and socioeconomic status.

One limitation is the lack of information on indication for SSRI. SSRI can be prescribed for depression as well as for other conditions such as anxiety, panic attacks, and obsessive compulsive disorders, and these different conditions may carry different suicide risks. There is an inherent risk that the symptoms of the underlying disease for which the SSRIs are meant to be treating might be unstable over time, and if so confounding the association. A meta-analysis on the effect of anti-depressants on suicide risk found substantially lower risk increase in trials for non-psychiatric indications and, hence, concluded depression to play a key role in suicidality and that antidepressants themselves did not generate additional suicidal symptoms [19]. Other studies show that 30-40 percent of DSM-IV defined unipolar major depressive disorder (MDD) patients have clinically significant hypomanic symptoms. Antidepressants can worsen the depression in unrecognized or covert bipolar depression via inducing mixed states by increasing manic symptoms. The clinical picture of this is very similar to activation syndrome [20,21]. However, by the possible inclusion of other conditions than depression, with inherent lower risks of suicide and without risk for activation syndrome, we expect our findings to be an underestimation of the true effect of the suicide risk during initiation of SSRI therapy in depression.

It is likely that SSRI therapy is initiated in patients that have an inherent increased risk of suicide, e.g. severe depression. Our main limitation is thus confounding by indication, where higher risk of suicide deaths during time periods with SSRI may depend on fluctuations in severity of the underlying depression that is related to SSRI-use rather than to the SSRI-use in itself. It is not possible to establish to what extent confounding by indication affects our overall risk estimates. In an attempt to explore this confound we performed the same study with TCAs instead that may be less likely to produce an “activation syndrome”. Since we obtained an OR of 2.00 [0,75-,5,33] for TCA (during the same time period 2007-2010) these results could be interpreted as slightly stronger evidence in favor of a serotonergic mechanism involved in at least a small subset of suicides. However, the descriptive question on whether there is an increased risk of suicide in the early phase of treatment, which is of great clinical relevance, is not confounded by indication. The bias only interferes with our understanding of the causation of this increased risk. However, the peak in the risk estimate in the second and third week after initiation in part could speak against confounding by indication, as the suicide risk rather would be anticipated to decrease with time. Also, the suicide peak in day 8 to 11 and in day 12-15 argue both in favor of the activation syndrome theory and of the biological mechanism through alteration in serotonin levels [13].

We avoided seasonal effects by choosing the control period 364 days prior to the suicide [22]. This approach also ensures that the index date in the control period occurs on the same week-day as the suicide. Despite this, we cannot rule out the effect of potential differences in the severity of the underlying psychiatric disease in the compared time intervals from a potential triggering effect of SSRI initiation, as discussed above.

Although SSRIs are the first-line treatment for major depression, 30–40% of the patients do not show a significant response [23]. If so we might overestimate the risk since some of the suicides might be due to lack of response rather than due to a biological mechanism from SSRI or activation syndrome. However, a possible lack of response would most probably not change over time, which leads us to believe it is not a major problem in our study.

To avoid changes and trends in classification we included deaths coded as undetermined intent which is common when studying suicide in Europe [15]. Our subdivided analysis showed however higher ORs among certain suicides compared to undetermined intent. The most common (around 70%) suicide method among deaths coded as undetermined intent is poisoning (over-dose), i.e. non-violent, whereas among certain suicide around 80% are violent (data not shown).

We regarded a dispensed prescription of SSRI within 28 days of index date and no dispensed prescription of SSRI in the preceding 4 months as being exposed to SSRI initiation. However, there was no information on when or whether the patients had actually taken their medication, which is a limitation. This potential misclassification of the exposure would, however, most likely be non-differential, i.e. independent of future suicide or not. Hence, we anticipate the same misclassification in the case and the control window, and this would thus only dilute the risk estimates towards null results.

The so called activation syndrome, a side effect of antidepressants, has been suggested to carry a potential for increased suicide risk [6,10]. It is however difficult to distinguish activation syndrome from worsening of a present psychiatric illness. It has also been stated that certain subgroups of patients, such as those with borderline personality disorders, are especially vulnerable to this syndrome [24]. Our study did however not include information on previous psychiatric diagnoses. In a case-control study of 555 cases and 2 062 controls, suicidal behavior increased in the first month after starting SSRI therapy, and especially during the first nine days which corresponds quite well to the suicide peak showed in our study [7].

We found the highest risk estimates among those aged 65 and older. Previous studies have shown higher suicide risks among adolescence [5,9,12]. Since we had too few exposed adolescence we could not look at this age group separately. However our youngest age group 13-25 did not display as elevated risk estimates as the other age groups. Our result indicates that the incidence of activation syndrome does not seem to be related to age [6].

There is evidence that biological mechanisms partly are responsible for impulsivity and aggression, which often coexist with suicidal behavior [13,25–27]. A matched case-control study found significantly reduced serotonin transporter availability in individuals with impulsive aggression compared with healthy subjects [28]. These findings could possibly contribute to explain our results showing an initial increased suicide risk during initiation with SSRI therapy.

Bridge et al. stated that treatment with antidepressants only makes sense in the context of education, continued clinical monitoring, and a viable safety plan which is well in line with our results [29]. It is important to stress that our results do not dissuade from prescribing SSRI. There is on the contrary evidence that this medication is helpful in depressed patients [1,3]. Since depression has been shown to play a key role in suicidality, every attempt to treat depression is therefore essential in a suicide prevention perspective.

To the best of our knowledge this is the first register-based study designed to explore the short-term risk of suicide after SSRI initiation. In conclusion, this large and nationwide study reports a peak in suicide risk during the second and third week of SSRI initiation. Together with a plausible biological mechanism and previous clinical and epidemiological observations our findings, linking initiation of SSRI to increased short-term suicide risk, deserve further attention. Extra attention to signs of suicidality is especially called for in the clinical setting and in the monitoring of patients during initiation with SSRI therapy. However, the effect could be present with other treatments for depression, and therefore the specificity of this effect needs to be studied further.
